# Recurrence and external sources differentially shape network correlations

**DOI:** 10.1186/1471-2202-14-S1-P113

**Published:** 2013-07-08

**Authors:** Moritz Helias, Tom Tetzlaff, Markus Diesmann

**Affiliations:** 1 Inst. of Neuroscience and Medicine (INM-6) and Inst. for Advanced Simulation (IAS-6), Jülich Research Centre and JARA, Jülich, Germany; 2 Medical Faculty, RWTH Aachen, Aachen, Germany

## 

The presence of correlated neuronal activity as such is not surprising, but rather a natural consequence of network connectivity and dynamics, in particular direct synaptic connections and shared local and non-local presynaptic sources [1]. The intriguing feature, however, is the modulation of correlations in relation to behavior [2]. These task-dependent changes may indicate that correlated spike timing is used for the storage, transmission, and processing of information. Moreover, correlated synaptic activation strongly influences the power and the spatial reach of the local field potential [3], a commonly recorded signal in experimental neuroscience.

A theoretical understanding of correlations requires the representation of (1) the recurrent connectivity and (2) external and internal sources of temporally varying or fluctuating signals. Such a theory is currently lacking. In particular it is still unclear how the recently found mechanism of active decorrelation [4], explained by negative feedback on the population level [5], affects the network response to externally applied stimuli. We here extend the theory of correlations in binary networks [6] to external correlated input. We show that (1) for homogeneous external input, the structure of correlations [7] is mainly determined by the local recurrent connectivity, (2) common external inputs provide an additive, homogeneous contribution to the correlation, (3) inhibitory feedback effectively decorrelates neuronal activity, even if neurons receive identical external inputs, and (4) connectivity with approximately identical synapses to excitatory and inhibitory cells increases internally generated fluctuations and pairwise correlations. We further present a new method which self-consistently includes correlations into the established mean-field description to increase the accuracy of the theoretical predictions. As a byproduct of our derivation we show that the cancellation of correlations between the summed inputs to pairs of neurons, in contrast to previous reports [4], does not uniquely determine the structure of correlations, but rather provides only a constraint, which is equivalent to the suppression of fluctuations on the population level [5]. We hope that our work provides a first stepping stone in the endeavor to understand the transformation of impinging correlated external activity into correlated activity between the neurons of the local network.

## References

[B1] Gerstein GL Perkel DH Science 1969 881 164 828 830 5767782 10.1126/science.164.3881.828

[B2] Kilavik EB Roux S Ponce-Alvarez A Confais J Gruen S Riehle A Journal of Neuroscience 2009 29 12653 12663 10.1523/JNEUROSCI.1554-09.2009 19812340 PMC6665105

[B3] Lindén H Tetzlaff T Potjans TC Pettersen KH Grün S Diesmann M Einevoll GT Neuron 2011 72 5 859 72 10.1016/j.neuron.2011.11.006 22153380

[B4] Renart A de la Rocha J Bartho P Hollender L Parga N Reyes A Harris KD Science 2010 327 587 590 10.1126/science.1179850 20110507 PMC2861483

[B5] Tetzlaff T Helias M Einevoll G Diesmann M PLoS Comput Biol 2012 8 8 e1002596 10.1371/journal.pcbi.1002596 23133368 PMC3487539

[B6] Ginzburg I Sompolinsky H Phys Rev E 1994 50 4 3171 3191 10.1103/PhysRevE.50.3171 9962363

[B7] Gentet L Avermann M Matyas F Staiger JF Petersen CCH Neuron 2010 65 422 435 10.1016/j.neuron.2010.01.006 20159454

